# Advances in the Research and Development of Natural Health Products as Main Stream Cancer Therapeutics

**DOI:** 10.1155/2015/751348

**Published:** 2015-03-26

**Authors:** Pamela Ovadje, Alessia Roma, Matthew Steckle, Leah Nicoletti, John Thor Arnason, Siyaram Pandey

**Affiliations:** ^1^Department of Chemistry & Biochemistry, University of Windsor, Windsor, ON, Canada; ^2^Department of Biology, University of Ottawa, Ottawa, ON, Canada

## Abstract

Natural health products (NHPs) are defined as natural extracts containing polychemical mixtures; they play a leading role in the discovery and development of drugs, for disease treatment. More than 50% of current cancer therapeutics are derived from natural sources. However, the efficacy of natural extracts in treating cancer has not been explored extensively. Scientific research into the validity and mechanism of action of these products is needed to develop NHPs as main stream cancer therapy. The preclinical and clinical validation of NHPs would be essential for this development. This review summarizes some of the recent advancements in the area of NHPs with anticancer effects. This review also focuses on various NHPs that have been studied to scientifically validate their claims as anticancer agents. Furthermore, this review emphasizes the efficacy of these NHPs in targeting the multiple vulnerabilities of cancer cells for a more selective efficacious treatment. The studies reviewed here have paved the way for the introduction of more NHPs from traditional medicine to the forefront of modern medicine, in order to provide alternative, safer, and cheaper complementary treatments for cancer therapy and possibly improve the quality of life of cancer patients.

## 1. History of Natural Health Products (NHPs) in Cancer

Natural health products (NHPs) and natural products (NPs) play a leading role in the discovery and the development of drugs for the treatment of human diseases. Traditional medicines in the Native American, Chinese, and Indian cultures have utilized numerous natural products, including dozens of spices and plant extracts. Scientific research into the validity of these traditional products has shown that many do indeed have potent anticancer effects [1, 2]. An extract from the Mayapple,* Podophyllum peltatum*, was traditionally used by Native Americans to combat skin cancers and other malignant neoplasms, as well as a host of other ailments. The major component of this extract was podophyllotoxin, which was the first in a series of effective anticancer agents called podophyllins [3]. Likewise, numerous natural products used in traditional Indian Ayurvedic medicine have been shown to have strong anti-inflammatory and anticancer properties.

**Figure 1:**
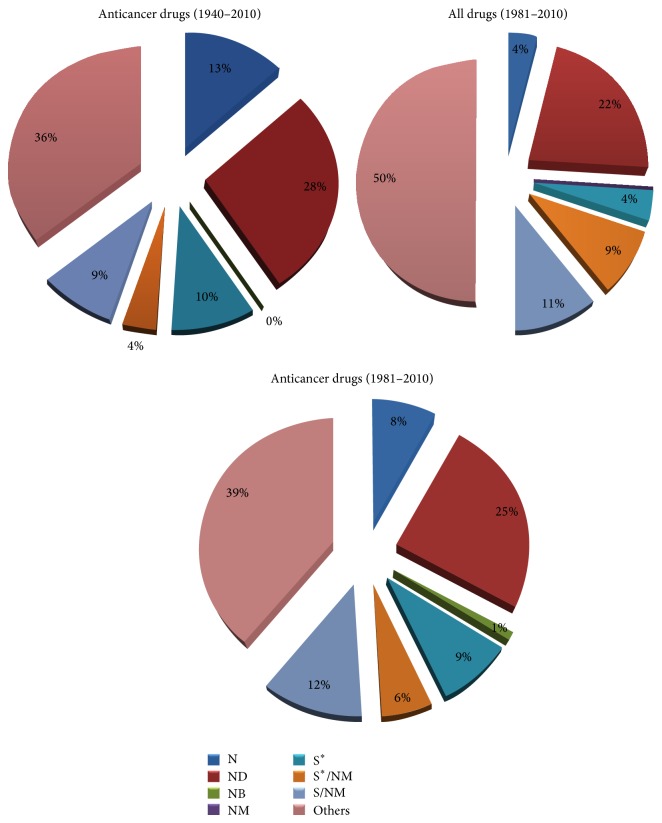
Sources of anticancer drugs from the 1940s to 2010. ^∗^Natural product (N), derived from a natural product, usually a synthetic derivative (ND). Natural product “Botanical” (NB); natural product mimic (NM); totally synthetic drug (S) made by total synthesis, but the pharmacophore is/was from a natural product (S^∗^).

Curcumin, the active ingredient in turmeric, has been widely studied for its anticancer properties. Turmeric (*Curcuma longa*), itself, was widely used in Ayurvedic medicine and the therapeutic benefits, which are now attributed to the presence of curcumin, include the ability to suppress tumor growth in a wide variety of cancer types [4, 5]. A total of 27 anticancer drugs from 1940 to 2010 were obtained from natural sources, for instance actinomycin D, paclitaxel, and vincristine, now one of the most commonly used chemotherapy agents in cancer treatment, while topotecan HCl, dexamethasone, etoposide, and even tamoxifen are mimics of natural products [1] (Figure 1). Camptothecin, found in extracts from* Camptotheca acuminate* and used in traditional Chinese medicine, has been found to have antitumor activity and its derivatives, topotecan, and irinotecan are routinely used to treat ovarian and colon cancers [6].

The discovery of the anticancer activities of so many traditional medicines and natural products has been supported by scientific evidence and validation. This was in part successful due to the initiation of the Cancer Chemotherapy National Service Center (CCNSC) in 1955, by the National Cancer Institute (NCI). The mandate of this program was to screen for antitumor agents on a larger scale by establishing a strict standardized protocol for testing potential anticancer compounds [7].

Since the 1980s, research into the anticancer effects of natural products has yielded many promising results. For example, resveratrol, a polyphenol present in grapes, shows potential as both a preventative and an antitumor agent [8]. Similarly, piperlongumine, extracted from* Piper longum*, selectively induces reactive oxygen species in cancerous cells, leading to apoptotic cell death [9]. In the 1980s, Bagshawe et al. developed a novel use for natural products, the antibody-directed enzyme-prodrug therapy (ADEPT). This technique used tumor-specific antibodies bound to an enzyme that would convert noncytotoxic prodrugs into their cytotoxic forms once in contact with the tumor [10, 11]. Many natural products were successfully used as prodrugs, including doxorubicin and Taxol [3]. These earlier studies have paved the way for the introduction of more NHPs from traditional medicine to the forefront of modern medicine. The scientific validation of these NHPs in terms of their efficacy, safety, and mechanism of actions will seal their position in modern medicine, especially in the field of cancer research and therapy.

## 2. Current Trends in NHP Research and Cancer

Even with all the incoming evidence, herbal drugs and other NHPs and NPs are usually shunned during systemic chemotherapy because of herb-drug interaction and exaggeration of chemotherapy-related toxicity. Current research is focused on the development of new and more effective chemotherapeutic agents that have little to no associated toxicity to the patient. Lately, this focus has been centered on NHPs and herbal formulations, mainly in the form of plants and other biological sources around the world. NHPs have been used for centuries by a variety of cultural backgrounds for a great number of illnesses; some of which continually provide new medicinal applications and intriguing anecdotal evidence, which merits further investigation. Today, there are numerous natural health products that fall under the umbrella of traditional medicine, such as the Indian herbs Tulsi (*Ocimum sanctum*), Neem (*Azadirachta indica*), and Ashwagandha (*Withania somnifera*), commonly known as Indian ginseng or winter cherry. These herbs have shown an incredible diversity of treatments for diseases in both ancient and modern times as well. Ayurvedic medicine has been very informative in the introduction of numerous NHPs. Tulsi, also referred to as “Holy Basil,” has in past decades been studied for its many health benefits, which includes but is not limited to treatments for bronchitis, pain, malaria, asthma, arthritis, cancer, diabetes, and numerous microbial infections [12, 13]. One study claims that it is primarily the phenolic compound, eugenol, to which the health benefits of Tulsi are owed [12]; however more recent research suggests that there is an additional range of compounds at work, including the phytochemicals rosmarinic acid, apigenin, myretenal, luteolin, *β*-sitosterol, and carnosic acid; all of which have been shown to be valuable in the reduction of chemically induced cancers through initiating apoptosis and maintaining antioxidative and antiangiogenic effects [14]. On the same page, Neem leaves have been shown to possess a strikingly similar range of pharmacological effects to Tulsi and in one study is referred to as a “living pharmacy” in itself [15]. The benefits of Neem range from reductions in inflammation, microbial infection, progression of diabetes, oxidative stress, cancer proliferation, and tumor development, indicating chemopreventive benefits. Some of the active compounds within Neem are Azadirone, Nimbidin, Nimbolide, and the Polysaccharides GIa and GIb [15, 16]. Ashwagandha has been a staple in traditional Indian medicine for decades and has been widely used, owing to the various properties that have been attributed to it. Ashwagandha is proposed to have antioxidant, anti-inflammatory, anticancer, antistress, and adaptogenic properties [17, 18]. The extracts of this plant have been studied intensely to validate the claims that have been the backbone of its use in ayurvedic medicine. A recent study in 2013 showed the efficacy of* Withania* extract against metastatic breast cancer. The ethanolic extract of this plant was efficient in preventing the invasion of breast cancer cells in a spheroid invasion assay, while inhibiting the metastasis of breast tumors to the lungs and lymph nodes in animal models [19]. In Phase II clinical studies, this herb was shown to promote “general well-being of patients,” when used in combination with chemotherapy, as well as enhance the cytotoxicity of chemotherapy in breast cancer patients. This combination treatment led to an increase in the quality of life of the breast cancer patients in this study [18, 20].

Another example of an NHP that has been used for centuries is the extract of dandelion, a perennial weed known for its curative properties. The dandelion species have been used in many traditional and modern herbal medicinal systems and this use has been documented all across the continents. Various parts of this plant have been used in the treatment of different ailments, with the root being used in gastrointestinal diseases and the leaves as a diuretic and digestive stimulant. The whole plant has been taken as a cure for hepatitis and anorexia as well, although some of the claims associated with this weed have gone unsubstantiated [21, 22]. Some preclinical research on dandelion has introduced this plant with numerous properties to the scientific community. Research has shown the anti-inflammatory, prebiotic, antiangiogenic, and antineoplastic properties of dandelion root [21]. However, some studies do not agree with others, leading to the publication of conflicting reports on this NHP. More recently, studies have shown selective efficacy of dandelion root extract (DRE) against several cancer cell types in a dose and time dependent manner. The investigation of the mechanism of action of dandelion root extract in cancer cells is under study, with focus on the identification of the possible apoptotic pathway in which this extract is selective to cancer cells. It has been shown that DRE targets the death-receptor mediated extrinsic pathway of apoptosis and its mechanism is dependent on the activation of caspase-8 [23–25]. Overwhelming scientific evidence with the aforementioned NHPs are paving the way for other NHPs, especially in cancer treatment and introducing these compounds and products as safe alternatives and effective contenders in the fight against cancer.

## 3. Mechanistic Efficacy of NHPs against Cancer

The different hallmarks of cancer and tumor cells include evading growth suppression signals, evading programmed cell death processes, inducing angiogenesis, and sustaining proliferative signaling, to name a few [26, 27]. These hallmarks have been studied in great detail and therefore provide multiple means to target cancer cells selectively. The study of NHPs against cancer cells and xenograft models has therefore focused on identifying NHPs that can target pathways that convey survival protection to cancer cells, so as to selectively and effectively eradicate cancer cells. This review focuses on the role of NHPs in several pathways that are involved in cancer, including inflammation and inflammatory response, oxidative stress, and mitochondrial response as well as receptor agonists/antagonists. The studies and results presented in this review are meant to highlight the importance of NHPs in the targeting of multiple pathways that are involved in cancer initiation and progression.

### 3.1. Natural Health Products in Inflammation and Inflammatory Response in Cancer

It is well-known that the inflammatory response is vital in living organisms for their protection against foreign matter. Inflammation more commonly occurs in cases of infection and injury (acute inflammation), but in certain situations, a more persistent, deregulated, and maladaptive inflammation (chronic inflammation) can arise. This chronic form of inflammation is usually associated with chronic diseases like cancer, where there is exacerbation of the disease, due to the prolonged inflammatory response. This would lead to increased proliferation of the cancer cells, increased angiogenesis, and promotion of metastatic capabilities [28, 29], making chronic inflammation a hallmark of neoplastic transformation [30]. Unlike acute inflammation, there is not much known about the processes and molecular pathways associated with chronic inflammation [29]; however, there are anti-inflammatories available that might be able to target the inflammation and possibly the molecular pathways involved in order to reduce the extent of inflammation and possibly the unwanted effect of inflammation in chronic diseases, like cancer. One of the most common examples for the role of inflammation in cancer progression has been the use of nonsteroidal anti-inflammatory drugs (NSAIDs) and COX-2 specific inhibitors, to reduce the risk of developing some cancers and preventing the mortality associated with those cancers [29]. These NSAIDs and COX-2 inhibitors act by interfering with eicosanoid signaling and metabolism, suppressing the formation of tumors and acting as antioxidants and antiangiogenics.

It has been found that there is a shared pathology between cancer and inflammatory diseases, which is displayed in the gene expression signatures for cancer and those for inflammatory diseases [30]. These findings suggest that targeting the inflammatory response is a potential way to target different forms of cancer. To combat unwarranted inflammation, anti-inflammatories like aspirin, ibuprofen, and prednisone are most commonly used, despite their side effects [31]. Alternatively, natural health products are used to heal a variety of ailments including inflammation in a safe and effective manner. This prompts continual research into the mechanism of natural anti-inflammatories as well as the discovery of new natural therapies. Several phytochemicals, including curcumin from turmeric and resveratrol from grapes, are used partly due to their anti-inflammatory activity. They inhibit inflammation by suppressing the activity of NF-*κ*B and possible STAT-3 [30, 32].

Long pepper or* Piper longum* L. has been used as both a spice and a therapy for a number of centuries. Historically, it was used as a topical treatment for muscle inflammation but has shown efficacy in a number of diseases and conditions including diabetes, cancer, and obesity without having any toxic effects [33]. More recently, the plant has been studied as an anti-inflammatory agent for carrageenan-induced paw edema in rats. In the study, researchers found a significant decrease in paw inflammation of rats treated with long pepper indicating that long pepper suppressed acute and subacute inflammation [34]. In addition to this study, other work has been done on piperlongumine, an important component of the long pepper fruit, as a therapy against atherosclerosis. This study found that the anti-inflammatory and antiplatelet aggregation properties of piperlongumine prevented artherosclerotic plaque formation in mice, proving it to be a possible therapy for this inflammatory disease [28, 35]. Furthermore,* in vitro* studies in various cancer cell lines showed the anticancer effect of piperlongumine in these cells, where it was shown that this compound was able to target the oxidative stress response of these cells, increase the levels of reactive oxygen species (ROS), and activate the expression of several key proapoptotic proteins. This anticancer effect was confirmed in* in vivo* models of breast adenocarcinoma [9]. Piperlongumine represents one of the compounds present within long pepper that provides the anti-inflammatory response from this plant; however the effect of this compound alone is significantly less than the whole plant extract in reducing inflammatory response [35, 36].

In addition to long pepper and piperlongumine, a natural compound found in grapes, peanuts, and berries known as* resveratrol* (3,4′,5-trihydroxystilbene) is a fat soluble compound that has also shown anti-inflammatory potential. Researchers became interested in elucidating the potential health benefits of resveratrol when it was reported to be present in red wine, which had previously been shown to reduce coronary heart disease by 20–30% [29, 37]. It has been found that resveratrol can have a direct effect on the immune response of the body and thus can be used as an immunomodulator in patients with inflammatory diseases [29]. More specifically, resveratrol suppresses the expression of inflammatory biomarkers like TNF, COX-2, iNOS, and CRP, preventing inflammation [30]. Additionally, resveratrol has been shown to impede the activity of at least one type of matrix metalloproteinase enzymes which assist the invasion of normal tissue by cancer cells. The anti-inflammatory properties of resveratrol have also been demonstrated* in vitro.* Studies are inconclusive on whether or not high intakes of resveratrol are effective in protecting against and preventing cancer in humans [38–40]; however, due to the ability of this compound to act as a potential therapeutic measure for cancer, it is essential to pursue further studies on this NHP. As noted earlier, due to the low bioavailability of resveratrol in humans, studies suggest that even high intakes of resveratrol may not result in the same anticancer effectiveness that was demonstrated in cell culture [41].

Dandelion extracts have been found to have anti-inflammatory activity in some cancer cells [22]. In a study by Jeon and colleagues in 2008, an ethanolic extract of the whole plant was shown to possess anti-inflammatory properties. This extract led to the downregulation of the production of NO and COX-2 in activated macrophage cells [42].

These findings suggest a great potential for NHPs with anti-inflammatory activity in the fight against cancer. The ability of these NHPs to target multiple pathways in inflammation and in cancer progression provides a potentially more efficacious way to selectively target cancer cells.

### 3.2. Natural Health Products in Oxidative Stress Response and Mitochondrial Involvement in Cancer

It has long been known that the mitochondria play a significant role in the carcinogenesis and cancer progression [43–45]. There are a number of metabolic alterations that are associated with mitochondrial functions that can be used to differentiate cancer cell mitochondria from normal cell mitochondria. For instance, the activities of some enzymes that are required for oxidative phosphorylation are usually decreased in cancer cells, unlike normal cells. It is however essential to note that although there are a large number of differences between a cancer cell and a normal cell mitochondria, these differences are not common to all cancer cells [44]. Mitochondrial respiration is coupled with the production of ROS and under normal conditions these oxidative species are usually neutralized into harmless forms. Under abnormal conditions, there is a corresponding increase in ROS and oxidative stress, which leads to the mutations in both mitochondrial and nuclear DNA, damage to proteins and lipids, and resistance to apoptotic induction. Oxidative stress and the resulting mutations are typically at the basis of some malignant phenotype, as can be seen in cancers [44, 45]. It has been hypothesized that chronic inflammation (discussed above) is linked to oxidative stress and ultimately to carcinogenesis [44]. Data from a study carried out in 1994 showed that there was an increase in mitochondrial DNA mutations, which are typically associated with ROS production [46]. Furthermore, studies have shown some commonly known antioxidants, like vitamin E and some flavonoids present in natural extracts, can inhibit inflammation, thereby inhibiting carcinogenesis and progression of cancers [47, 48].

A lot of research has gone into antioxidant mechanisms and the roles they play in tumor development. Some of this work has provided better understanding of NHPs in oxidative stress response, especially in cancer development and treatment. There are increasing numbers of NHPs that are involved in oxidative stress response. One famous compound, piperlongumine, discussed above, has been shown to target and inhibit the endogenous oxidative stress response of cancer cells, leading to an increase in the levels of ROS and a corresponding increase in oxidative stress. The inability of the cells' oxidative stress response to detoxify these reactive oxygen entities led to the induction of apoptosis in cancer cells. This effect was countered by the presence of the antioxidant, N-acetylcysteine. More importantly, this effect on ROS generation and oxidative stress pathway targeting was not observed in the noncancerous cells, suggesting a dependence on the oxidative stress response pathway in cancer cells [9]. These results confirm what has been previously known: targeting the mitochondria could provide a better selective target in cancer cells [49], for more efficacious treatment.

Some NHPs are proposed to contain both antioxidant and prooxidant properties. Dandelion (*Taraxacum officinale*) flower ethanolic extract is considered one of such NHPs [50]. The purpose of this study was to characterize the antioxidant properties of dandelion flower extract (DFE), which was attributed to the presence of luteolin and luteolin-7-glucoside. Interestingly, it was observed that higher concentrations of this extract had prooxidant effects in colon cancer cells. This is an important finding as it shows the versatility of NHPs under different situations and conditions. It is also essential to note that the production of ROS can have both a proapoptotic and an antiapoptotic effect, depending on the conditions of the cells [51]. There is a growing body of evidence that proves that ROS production acts not only as destructive agents but also as chemical messengers [51]. This information, along with the fact that NHPs are so versatile, further proves the importance of NHPs as treatment options.

Lots of studies have been carried out with other NHPs; for instance, epigallocatechin-3-gallate (EGCG) has been studied for its antioxidant capabilities for decades and treatment with this compound significantly slowed down the growth of breast cancer tumors in mice [52]. Other nutraceuticals, like resveratrol (discussed above), also have beneficial claims in antioxidant therapy. Several studies have shown that NHPs rich in flavonoids and phenolics play a significant role in oxidative stress response and concomitant use of NHPs with these components increases the activities of the other NHPs [48, 52, 53].

There are a lot of characteristics attributed to curcumin and its role as an anticancer agent. Curcumin, from turmeric, is another natural compound that has both antioxidant and prooxidant characteristics and this capability has been studied in various cell culture models and confirmed in* in-vivo* studies [5, 54, 55]. These studies definitely speak to the versatility of NHPs and natural compounds, in targeting multiple cell pathways, especially in the fight against cancer.

Even with advances in NHP research, we are still a long way from understanding the connections between some of these NHPs and oxidative stress, especially in cancer research. It is therefore essential to further investigate how NHPs are able to distinguish between different conditions and act in accordance to both scavenge and induce the production of radical oxygen species.

### 3.3. Natural Health Products as Receptor Agonists and Antagonists in Cancer

Abnormal and excessive signal transduction is a common hallmark of cancer cells. This is in part due to the ability of these cell types to upregulate the expression of both receptors and the ligands (usually growth factors) required to transmit downstream signals. This ability thereby confers hyperproliferative characteristics to cancer cells; for instance, this is observed in aberrant Ras and myc signaling [56]. This therefore suggests that a way to target cancer cells effectively will be to target the aberrant signaling pathways, either by knocking down the expression of ligands and/or receptors or preventing signal transduction by introducing an antagonist, thus, indicating the importance of NHPs as potential anticancer agents. Several NHPs have been reported to play an antagonistic role against several important receptors in the aberrant signaling pathways in cancer cells. Due to the presence of many components in some of these NHPs, it is not surprising that one or more of these components could target different receptors and signaling pathways.

One particularly interesting class of compounds is the sesquiterpene lactones (SLs), found in an initial screen by the National Cancer Institute (NCI), the same screening that led to the identification of Taxol [57]. These SLs are generally plant secondary metabolites and although almost exclusive to the Asteraceae plant family, they can also be found in the Umbelliferae and Magnoliaceae families as well. These compounds are worth further investigation for their development as anti-inflammatory and selective anticancer agents [57]. Owing to this ability, extracts of plants high in SLs have been given considerable interest, especially in cancer and inflammatory diseases [23–25, 42, 57]. SLs have been shown to selectively target cancer stem cells and a host of them has successfully proceeded to Phase I and Phase II clinical trials. Some common ones include Artemisinin (*Artemisia annua* L.), Thapsigargin (*Thapsia*), and Parthenolide (*Tanacetum parthenium*) and these have been successful in a lot of studies involving various types of cancers: laryngeal, breast, colorectal, and non-small cell lung carcinoma (Artemisinin); breast, kidney, and prostate cancers (Thapsigargin); and AML and lymph node cancers (Parthenolide). The activity of these SLs is mainly attributed in part to their ability to target cell surface transferrin receptors (a distinct hallmark of rapidly proliferating cells), NF-*κ*B signaling (by disrupting the recruitment of I*κ*B kinase complex to the TNF receptor, which is essential for tumor initiation, progression and resistance), and the angiogenesis pathways, by inhibiting human vein endothelial cell proliferation and vascular endothelial growth factor and receptor expression [57, 58]. Antiangiogenic drugs have been of great interest, especially in the field of cancer. The generation of new blood vessels provides tumor cells with growth and survival advantage, as tumor cells depend on an adequate supply of oxygen and nutrients for continued survival. This increase in tumor vasculature also increases the chances of metastasis to distant sites. Therefore finding antiangiogenic drugs that could not only inhibit angiogenesis but also decrease the chances of tumor metastasis is of utmost importance. The efficacy and benefits of SLs as antiangiogenics are proof that this (angiogenesis) is an essential target in the fight against cancer. More recently, there have been increasing evidence for the role of NHPs as antiangiogenics, especially those containing bioactive phytochemicals like sesquiterpene lactones. Moreover, the fact that many NHPs contain multiple bioactive phytochemicals makes them a viable source for receptor agonists and antagonists. There are many reports of natural health products with direct and indirect effects on angiogenesis; see Table 1 [47, 59–65]. These NHPs act to target the different pathways involved in angiogenesis by decreasing the expression of target proteins and receptors. For instance, the epidermal growth factor and its corresponding receptor have downstream effects on urokinase-type plasminogen activator (uPA), which in turn can promote angiogenesis. In addition, COX-2 and lipoxygenase (LOX-5) tend to have stimulatory effects on cancer progression and angiogenesis and increase in COX-2 expression is associated with a progression to invasive phenotypes in certain cancer types. Furthermore, vascular endothelial growth factor (VEGF) is associated with increased proliferation and migration of endothelial cells and an increase in the expression of metalloproteinases (MMP), leading to increased vascularization within a tumor that promotes metastatic capabilities. These suggest that NHPs and NPs that can target the expression of key players in angiogenesis (such as those listed in Table 1) are viable options for the treatment of cancer.

Several other receptors play a role in the progression of cancers, especially the estrogen (ER) and androgen receptors (AR) which are seen in breast and prostate cancers [66]. There are two main isoforms of the ER, the alpha (ER-*α*) and the beta (ER-*β*) isoform, which have been shown to play opposing roles in the initiation and progression of breast cancers; the alpha isoform promotes tumor formation and progression, while the beta isoform takes on the role of a tumor suppressor [67]. This information indicates that these receptors are another viable option in the attempts to target cancer cell and tumor progression. Emerging research suggests that the activation of the AR is able to inhibit breast cancer progression [67], and its expression is a significant prognostic marker in estrogen receptor positive breast cancers [68]. This suggests that downregulating the expression of the ER and/or activating the downstream signaling of the AR can be essential in the fight against breast cancer; however, AR plays a significant role in the development and progression of therapy-resistant prostate cancer [69, 70], indicating that activation of AR to treat breast cancer will have dire effects on the prostate and suggesting that a focus on ER antagonists would be a more efficacious option. Lately, more research is going into identifying NHPs and NPs that can target these receptors, leading to a decrease in their downstream activity.

Perhaps the most common example of an ER antagonist is tamoxifen, which is used especially for hormone-receptive breast cancer treatment. Recent studies have shown that administration of soy isoflavones, such as genistein and daidzein, can have an effect on the efficacy of tamoxifen. Some studies suggest that some isoflavones (genistein) can increase the potency of tamoxifen in ER− breast cancer cells and have the opposite effect in ER+ cells, while others indicate that daidzein, in combination with tamoxifen, has increased protection against both ER+ and ER− breast carcinomas [71]. This indicates that the use of NHPs/NPs must be used with caution and advocates a necessity for scientific validation of the mechanism of actions, indications, and contraindications of NHPs/NPs, to ensure safety and lack of toxicity associated with the administered treatment.

Several NHPs have been shown to induce different types of programmed cell death, including apoptosis, necrosis, and autophagy, in cancer cells, some in a selective manner. However, understanding the mechanism of action of these NHPs sometimes proves difficult, as the multiple components tend to have multiple targets. This has led to increasing mechanistic studies into effective NHPs and some of these studies have identified active NHPs with receptor antagonistic activities. For instance, withaferin A, a naturally occurring bioactive component isolated from* Withania somnifera,* was shown to knock down the expression of ER-*α* but not ER-*β*, leading to its role in chemoprevention and apoptosis induction [72].

As mentioned earlier, several compounds found in soy isoflavones have significant anticancer activity, with genistein and daidzein's ability to target HER2/neu and EGFR, to inhibit angiogenesis [59]. Several studies have also shown that these compounds are able to target the ER, however not with great affinity. Another study showed that one of these compounds, daidzein, is converted to corresponding Equol by gut microflora. There are two isoforms of this compound: R-Equol, with a moderate binding preference for ER-*α* and S-Equol, with a strong binding preference for ER-*β* and both of these have much higher binding affinities for the ERs than their biosynthetic precursor compound, daidzein [73]. This suggests not only that NHPs contain bioactive components that could have multiple targets, but also that this bioactive components could act as precursor compounds for other compounds with even better activities against the different targets, as seen with the ER affinities of daidzein and its later compounds.

Another source of bioactive components is the long pepper, from the genus* Piper* (Piperaceae). These species are one of the most widely used NHP worldwide, with many biologically active secondary compounds having been identified in this species. The most common compound identified in piper spp. is piperlongumine (PL). Scientific evidence has shown the anticancer efficacy of PL by targeting the ROS stress response mechanism in cancer cells [9]. Further studies have also shown the ability of PL to target receptors, including the PDGF receptors for the inhibition of angiogenesis and more importantly treatment with this compound led to the depletion of androgen receptor in prostate cancer cells, through a proteasome-mediated ROS dependent pathway [74, 75]. This not only suggests the role of this NP as a receptor antagonist, but also provides a link between oxidative stress and receptor targeting, for an alternative path to cancer cell specific targeting, proving this usefulness of NHPs and NPs in the fight against cancer.

Aside from receptors involved with increased gene expression and cell growth and proliferation, death receptors are also key players in normal and cancer cell growth and survival. These receptors belong to the tumor necrosis factor (TNF) superfamily of receptors that play a role in signaling cell death and survival pathways and include TNFR1/TNF-*α*, FasR/FasL, and TNF-related apoptosis inducing ligand, TRAIL/TRAIL-R1 (DR4), or TRAIL/TRAIL-R2 (DR5) [76, 77]. These death receptors are ubiquitously and constitutively active in tissue types in the human body, with some tissues having a higher expression than others. The main difference between normal tissues and tumor tissues is the increased expression of the noncanonical prosurvival signaling of these receptors in tumor cells; for instance, many tumor cells overexpress TRAIL-R3 and TRAIL-R4 (decoy receptors, DcR1 and DcR2), with truncated cytoplasmic death domains that prevent the transmission of signals following binding of ligand to receptor. Also there is evidence that implicates several noncytotoxic pathways that are mainly facilitated by the activation of NF-*κ*B and MAPK pathways, through the activation of RIP kinase [77, 78]. This indicates that the death receptors provide another target in the fight against cancer. For instance, over the years, TRAIL and other ligands against TRAIL-R1/R2 have generated considerable interest, due to the selectivity of these ligands towards cancer cells, with little to no toxicity to noncancerous cells [77], suggesting their usefulness as cancer therapies.

Studies have shown that several NHPs are able to target death receptor signaling pathways, again confirming their usefulness as potential anticancer agents. The selective anticancer efficacy of dandelion root extract has been attributed to its ability to induce death-receptor mediated extrinsic apoptosis in cancer cells selectively [23–25], and a loss of this activity was observed in cells with a dominant negative Fas-Associated Death Domain (FADD) [24]. This knack for death receptor targeting can be attributed to the presence of sesquiterpene lactones [58] and the suppression of cellular FLICE-like inhibitory protein (cFLIP), which is highly expressed in several cancer cell types, including pancreatic cancer cells, by the triterpene, lupeol [79]. This compound is one of the bioactive components of dandelion extracts [23, 80]. This inhibition of cFLIP has been shown to render TRAIL-resistant cancer cells sensitive to TRAIL therapy [79]. A derivative of resveratrol was found to induce FADD-dependent apoptosis in several human leukemia cells, significantly higher than resveratrol by itself. This dependence on FADD was not contingent on the expression of Fas, TRAIL, or TNF-*α* [81], suggesting that even in the absence of ligand and/or receptor, some NPs could still activate death receptor extrinsic pathway to selectively target cancer cells to apoptosis.

Taxanes (e.g., paclitaxel and docetaxel), isolated from Taxus, are effective as cytotoxic agents, as they target and stabilize the microtubules and prevent their depolymerization thereby interfering with normal cell functions and induce apoptosis. Further studies on the mechanism of taxanes indicate that these compounds can induce the expression of TNF-*α* and decrease the expression of certain TNF receptors [52], where prodeath TNFR1 expression was decreased and prosurvival TNFR2 expression was increased [82]. This could suggest a way by which cancer cells develop resistance to taxane treatment, although further investigation into this mechanism is still required, as TNFR2 is also implicated in cell death signaling [82].

Curcumin, discussed in previous sections of this review, has shown significant anticancer activity in several cancer cells, with the ability to target multiple signaling pathways. Not only does it antagonize cell surface receptors, like the epidermal growth factor receptor (EGFR), but it has also been shown to induce apoptosis in human melanoma cells through the activation of the Fas receptor and subsequent activation of caspase-8 [83], proving itself a worthy opponent in the fight against cancer progression.

These indications of the roles of NHPs and NPs as agonists and antagonists of receptors for the selective targeting of cancer cells to the process of cell death give further validation to the use of these products as safer alternatives to current cancer treatments. However, these examples also indicate that there is a lot of work that is required to further understand how these NHPs/NPs are able to recognize the aberrant signaling system in cancer cells and exploit these differences and vulnerabilities, although these studies provide a stepping stone for future validation of NHPs in the mechanistic validation of selective targeting of cancer cells for programmed cell death processes.

## 4. Characterization of Complex Natural Health Products: Fractionation and Metabolomics Profiling of Active Fractions

NHPs are usually complex mixtures that contain many bioactive substances. Dosage forms are difficult to characterize, as traditional preparation, dosage, and usage do not account for the presence of the various bioactive components. It is therefore imperative that identification of pharmacologically active ingredients within any NHP is carried out. As mentioned earlier,* in-vitro* studies of dandelion root extract have shown its efficacy in various cancer cell lines, giving it the potential to be developed as an anticancer agent. Dandelion root is a very complex biological material containing many bioactive substances and there are several ways of preparing dandelion roots for consumption. Infusions or decoctions of dry roots have been used by cancer patients in case report of individuals who have gone into remission. To provide a reproducible dosage form, a highly standardized decoction of roots can be prepared and lyophilized for clinical and phytochemical evaluation. The resulting standardized product, from dandelion root, is a brown powder which was reconstituted in water and polysaccharides precipitated by 1 : 1 addition of ethanol, using a procedure adapted from purification of ginseng polysaccharides. The resulting polysaccharide fraction was obtained by centrifugation and represents 20% by weight of the product. This material is mostly inulin, a nondigestible polymer of glucose, rather than starch found as a storage product in many plants. As well small amount of plant cell wall derived hemicellulose and pectin are extracted. While these substances have yet to be assayed, inulin is generally considered to be relatively inert, while cell wall derived polysaccharides may have immunostimulant activity, as has been found for ginseng acidic polysaccharides that act via toll-like receptors. The remaining 80% of the products are small molecular weight compounds, which were subjected to metabolomic analysis with a Waters Xevo UPLC MS QTOF. This fraction is highly complex and 91 compounds were selected for identification based on published literature. Identification of 14 compounds was achieved by elemental composition and monoisotopic mass observed following electrospray ionization. The compounds identified are found in Table 2 and, except for sucrose, they represent known or potentially bioactive phytochemicals. Many of the compounds are sesquiterpene lactones, such as taraxifolide or phenolic glycosides such as cichorioside.

Using standard pharmacognosy methods for the identification of active principles, identification of active principles can be achieved. These studies provide the backbone that is required to ensure standardization of NHPs that could be beneficial in the treatment of diseases, especially cancer.

In conclusion, antiproliferation activity has been found in both (water) polar and nonpolar (ethanolic) fractions of many NHPs, for instance, with dandelion root extracts, which contain sesquiterpenes, phenolics, and triterpenes [42, 50]. As with many medicinal plants activity may be connected with the joint action of several compounds rather than a single active molecule. Further work is required to assay the antiproliferative activity of the identified compounds and examine their combined activity.

## 5. Combinatorial Activity of Natural Health Products

With all the evidence put forward in the previous sections of this review, it stands to reason that the multiple components present within an NHP play a role in and are responsible for the selective efficacy of these NHPs against cancer cells,* in vitro* and in xenograft models. It is therefore essential to carry out further studies on a whole extract of an NHP to determine if it has better efficacy as a whole or if the isolated and characterized compounds provide the efficacy and selectivity we require for cancer treatment. Studies on* withania somnifera* have led to the identification of several bioactive components (e.g., the efficacy of withaferin A); however studies, including clinical trials, have shown that the effect of singular compounds found within this extract does not compare to the benefits of using the whole extract, especially as an anticancer agent [17–20]. Unpublished data with some of the identified bioactive phytochemicals in dandelion root extracts also indicate that that the efficacy of the whole extract or a combination of two or more bioactive components is better than each of the single compounds themselves. Traditional medicine practice has been known to combine multiple NHPs for better advantage [60, 84]. These combinations might also prove to be more selective than single component treatments. As seen with most of these NHPs that are able to target multiple signaling pathways, these characteristics are mainly due to the presence of multiple components. It is possible that the precise combinations of these components prevent or decrease toxicity associated with treatment, while proving to be more efficacious, at lower treatment doses, due to the synergistic activity of the different compounds. Hence NHPs appear to provide an alternative to current chemotherapy by providing safer, lower dose treatment options or providing a source of NPs that can be garnered for cancer treatment. Furthermore, a combination of two or more NHPs might increase the efficacy and selectivity, while reducing the chances of developing resistance to treatment, as these multiple NHPs could target even more pathways and be used at even lower doses.

Extensive scientific validation will be required to determine the efficacy, safety, and mechanism of action of the combined treatment options for the effective treatment of cancer. The effectiveness of these NHPs may be increased when multiple agents are used in optimal combinations.

## 6. Health Agencies and Regulatory Bodies Involved with Natural Health Products

The whole purpose of the scientific validation of NHPs against diseases, especially cancer, is to provide awareness for these NHPs and NPs that have been used for centuries in various traditional medicines. The scientific studies carried out provide the necessary evidence regarding the efficacy of these NHPs, their indications and contraindications, and information on their safe and effective use. In Canada, Health Canada is the governing agency for the introduction of drugs and NHPs to the public, with divisions completely dedicated to NHPs, the Natural Health Product Directorate (NHPD), and the Therapeutic Product Directorate (TPD). These divisions were generated to assist and ensure that Canadians have access to NHPs that are “safe, effective, and of high quality, while respecting freedom of choice and philosophical and cultural diversity.” Regulations for NHPs came into effect in 2004 and take into account their unique nature and characteristics. At the end of 2012, the NHPD published information that outline how NHPs are assessed, with a focus on health claims, the use of risk information, and the use of NHPs in combination; these include the “Pathway for Licensing NHPs Making Modern Health Claims”, “Pathway for Licensing NHPs making Traditional Health Claims” and “Quality of Natural Health Products Guide”, which summaries the requirement for standardization of high quality NHPs. Even after clinical trials and progression to the market, Health Canada continues to collect information on adverse reaction reports for NHPs, to track and analyze these reaction reports for NHP use through the Canada Vigilance Program and other regulatory agencies, like the World Health Organization (WHO). This allows constant monitoring of NHPs to ensure continuous safety and efficacy associated with these forms of treatment. More information on application to Health Canada and requirements involved in getting an NHP to the market can be found at their website: http://www.hc-sc.gc.ca/dhp-mps/prodnatur/index-eng.php.

In the United States, the Food and Drug Administration (FDA) is the agency in charge of regulating the production and provision of NHPs to the public. NHPs are referred to as complementary and alternative medicine (CAM), which are divided into 5 main domains:

- whole medical systems; Ayurveda, homeopathic medicine, and traditional Chinese medicine (TCM). This is the most common domain of NHPs/CAMs, which requires vigorous reviews and validation by the scientific community,
- mind-body medicine; meditation, prayer, and creative therapies, such as dance,
- biologically based practices with herbs, foods, vitamins, and dietary supplements,
- manipulative and body-based practices; chiropractic and osteopathic manipulation and massage,
- energy medicine, including therapeutic touch.

These domains undergo the same levels of rigorous review, as described in the Health Canada review aspect above. More information on application to the FDA and their requirements can be found at their website: http://www.fda.gov/regulatoryinformation/guidances/ucm144657.htm.

These regulatory agencies ensure that health claims made by traditional medicine have scientific validations for anecdotal evidence presented for centuries. They ensure proper standardizations involved in the production and usage of NHPs/NPs/CAMs to maximize the benefits of these products and medicines.

## 7. Significance and Conclusions

The toll of cancer on the human body and the society as a whole indicates a serious need for a better selective, effective, and cheaper mode of treatment. Natural health products hold a great potential to provide nontoxic alternatives for the treatment of cancer. More importantly, NHPs as a complex polychemical mixture of pharmacologically active compounds may target multiple vulnerabilities of cancer cells, without toxicity to the noncancerous cells. The complete scientific and clinical evaluation of the potential NHPs are essential to bring these products to mainstream cancer therapies, in order to provide alternative, safer, and cheaper complementary treatments for cancer therapy and possibly improve the quality of life of cancer patients. With the health regulatory agencies, including Health Canada and the FDA providing the required regulatory framework for the development of NHPs for therapeutic purposes, the future will see the growth and expansion of many cancer-selective NHPs in mainstream cancer treatment.

## Figures and Tables

**Table 1:** Natural health products that target the angiogenesis pathways.

NHPs/NPs	Target (decrease)
*Artemisia annua* (artemisinin)	VEGF/KDR
*Camellia sinensis* (epigallocatechin)	VEGF protein kinase C
*Chrysobalanus icaco* (methanol extract)	
*Curcuma longa* (curcumin)	MMP-2/9 VEGF bFGF CD13 (aminopeptidase N)
*Ginkgo biloba* (ginkgolide B)	VEGF
*Magnolia obovata* (honokiol)	PGDF TGF *β*
*Polygonum cuspidatum* (resveratrol)	MMP-2 VEGF
Quercetin	COX-2 lipoxygenase-5 enzymes EGFR
*Scutellaria baicalensis* (baicalin and baicalein)	VEGF bFGF 12-lipoxygenase activity MMP
*Silybum marianum* (silymarin)	VEGF
*Viscum album* (lectins)	VEGF
*Squalus acanthias* (dogfish liver: squalamine)	HER2
Soy isoflavones (genistein, daidzein)	EGFR (HER1)
*Aloe barbadensis* (aloe vera leaf and pulp extracts)	HER2/neu
Omega-3 fatty acids (eicosapentaenoic acid, docosahexaenoic acid)	COX-2 lipoxygenase-5 enzymes EGFR
*Ocimum sanctum* (carnosol, ursolic acid)	COX-2 NF-*κ*B several tyrosine kinases EGFR
*Rosmarinus officinalis* (carnosol and ursolic acid)	COX-2 NF-*κ*B several tyrosine kinases EGFR
*Zingiber officinale* (6-gingerol)	COX-2 NF-*κ*B

**Table 2:** Compounds identified in standardized GMP decoction of dandelion roots by UPLC MS QTOF.

Compound	Molecular formula	Monoisotopic mass
4-Hydroxybenzoic acid	C_7_H_6_O_3_	138.0316941
Sucrose	C_12_H_22_O_11_	342.1162116
Vernoflexuoside	C_21_H_28_O_8_	408.1784179
Pollinastanol	C_28_H_48_O	400.3705162
Austricin	C_15_H_18_O_4_	262.1205091
Lycoperodine 1	C_12_H_12_N_2_O_2_	216.0898776
11-beta, 13-dihydrolactucin	C_15_H_18_O_5_	278.1154237
Cichorioside C	C_21_H_32_O_9_	428.2046326
Taraxafolide	C_21_H_28_O_10_	440.1682471
Sonchuside A	C_21_H_32_O_8_	412.209718
Annuolide D	C_15_H_20_O_3_	248.1412445
Notoserolide A	C_21_H_28_O_9_	424.1733325
Taraxacin	C_15_H_14_O_3_	242.0942943
Taraxinic acid beta-D-glucopyranosyl ester	C_21_H_28_O_9_	424.1733325
